# Effect of Modification by β-Amylase and α-Glucosidase on the Structural and Physicochemical Properties of Maize Starch

**DOI:** 10.3390/foods13233763

**Published:** 2024-11-24

**Authors:** Xinge Jia, Jingwen Xu, Yan Cui, Dazhi Ben, Chuyu Wu, Jing Zhang, Mingru Sun, Shuo Liu, Tianhao Zhu, Jingsheng Liu, Ke Lin, Mingzhu Zheng

**Affiliations:** 1 College of Food Science and Engineering, Jilin Agricultural University, Changchun 130118, China; 2 National Engineering Research Center for Wheat and Maize Deep Processing, Changchun 130118, China

**Keywords:** starch modification, enzymatic modification, amylose/amylopectin ratio, α-1,6-glycosidic linkages, branching degree

## Abstract

Single enzymatic modifications are limited to starch. Complex modification with synergistic amylases will improve starch properties more significantly. In this study, maize starch was compound modified by β-amylase and α-glucosidase. The structure and physicochemical properties of the corn starch were determined by scanning electron microscopy (SEM), X-ray diffractometry (XRD), Fourier transform infrared spectroscopy (FT–IR), proton nuclear magnetic resonance hydrogen spectroscopy (^1^HNMR), high-performance anion-exchange chromatography (HPAEC–PAD), differential scanning calorimetry (DSC) and Rapid Visco analyzer (RVA) to determine the changes in the structure and physicochemical properties of maize starch before and after the dual enzyme modification. The branching degree (4.95–7.10%) of maize starch was increased after bi-enzymatic modification, the amylose content (28.77–18.60%) was decreased, and the amylopectin content (70.79–81.71%) was elevated. The relative crystallinity (20.41–30.20%) and short-range ordered structure of the starch increased, and the dual enzyme modification led to a more compact structure. Dual enzyme-modified maize starch showed a decrease in long chains, an increase in short chains, and its degree of branching was elevated. Dual enzyme modification also affected the thermal stability, pasting, light transmittance (1.40–2.16%), solubility (20.15–13.76%), and swelling (33.97–45.79%) of maize starch. It can be concluded that the complex modification of maize starch by β-amylase and α-glucosidase significantly changed the amylose/amylopectin ratio of the starch and made its structure denser. These results can provide a theoretical basis for the enzymatic preparation of maize starch with different amylose/amylopectin ratios and the development and utilization of functional starches.

## 1. Introduction

Starch is the primary source of carbohydrates in daily human life [1]. Natural starch comprises amylose and amylopectin starch; amylose and amylopectin have different structures and properties [2]. Changing the structure, chain-length, number of branching points, and amylose/amylopectin ratio can change starch’s internal structure, crystallinity, viscosity, digestibility, and other properties [3]. Therefore, the amylose/amylopectin ratio is an essential factor affecting its physicochemical properties, such as crystallinity, swelling power, pasting, and digestibility. Li et al. [4] used raw malt α-amylase to shorten the amylose and amylopectin length of maize, retard the regrowth of glutinous and common maize starch (NMS), and significantly reduce their pasting viscosity. Han et al. [5] prepared resistant starch from high amylose using hydrothermal, hydrothermal combined ultrasound, hydrothermal–alkaline, and thermal–alkaline combined ultrasound, which resulted in a significant increase in relative crystallinity, short-range ordering, and thermal stability.

In recent years, since the original properties of natural starch can no longer meet industrial demand, some modification methods have been applied to starch processing, and the modified starch produced is widely used in food, medicine, the chemical industry, and other fields [6,7]. Currently, the primary methods for producing modified starch are physical, chemical [8,9], and enzymatic [10]. Among these, enzyme modification has the advantages of specificity, environmental friendliness, and greenness, and it is the standard method of industrialized modified starch [11]. Enzyme modification modifies the internal structure of starch by utilizing the specific catalytic effect of amylase to improve some physicochemical properties of starch by changing the structure of starch molecules [12]. The specificity and exclusivity of enzyme modification minimize the generation of by-products, thus better obtaining final products with specific properties [13].

Due to the limited effect of single enzyme modification, the combined action of multiple enzymes enhances the modification effect on starch [14]. Li et al. [15] utilized the modification of maize starch by employing α-D-glucan branching enzyme and cyclodextrin glucosyltransferase and found that the dual-enzyme-modified starch had a higher content of slow-digestible and resistant starch compared to natural maize starch and single enzyme-treated starch. In the food industry, compound enzymes are often used to shorten the number and length of the outer chain of amylopectin starch or increase the branching density to change the amylose content of starch, and then change properties such as pasting, digestion, and rheology, to achieve the required characteristics [16]. For example, Yuan et al. [17] modified cross-linked tapioca starch with two α-amylases to improve its gel strength, using it as raw material. Ji et al. [18] used cyclo-dextranase and cyclodextrin glycosyltransferase to modify glutinous maize starch in order to change its molecular structure, oligosaccharide composition, and digestibility.

Diverse types of amylases have other effects, and complex modifications of two or more amylases with synergistic effects are more effective than modifications using a single enzyme. During the enzymatic production of maltose, β-amylase is the primary enzyme that cleaves explicitly α-1,4 glycosidic linkages and generates maltose units from the substrate non-reducing terminals [19]. β-Amylase shortens the length of the outer chain of the starch molecule and increases the short chains. α- Glucosidase (GT) effectively catalyzes the pasting of starch by hydrolyzing the α-1,4-glycosidic bond of the starch side chain to obtain a shorter chain-length [20]. It also transfers a free glucose residue to the starch chain in the form of an a-1,6-glycosidic bond, forming new branches and thus increasing branch density [21]. However, little information is available on the combined modification of maize starch using β-amylase and α-glucosidase. β-amylase, may have a synergistic effect with α-glucosidase, in which β-amylase cleaves the α-1,4 glycosidic bond and α-glucosidase increases the α-1,6 glycosidic bond, contributing to the formation of new branches, increasing the degree of amylose branching and decreasing the amylose content. In this study, we hypothesized that the synergistic action of β-amylase and α-glucosidase alters the ratio of amylose/amylopectin in starch, thereby affecting the macroscopic and microscopic structure of starch granules. We believe this will be of research value.

In this study, β-amylase and α-glucosidase were used to modify maize starch jointly to change the amylose/amylopectin ratio of starch and to investigate the effects of this change on its structural and physicochemical properties. This study can provide a theoretical basis for the enzymatic preparation of maize starch with different rectilinear/amylopectin ratios and a valuable reference for the further development of starch-based foods.

## 2. Materials and Methods

### 2.1. Materials

Maize starch came from Shanghai Ruiyong Biotechnology Co., Ltd. (Shanghai, China). β-amylase (BA, 50 U/mg) and α-glucosidase (GT, 700,000 U/mL) were purchased from Shanghai Yuanye Bio-Technology Co., Ltd. (Shanghai, China). All reagents used in this work were of analytical grade.

### 2.2. Methods

#### 2.2.1. Preparation of Maize Starch Modified by Dual Enzyme

Maize starch (20%, *w*/*v*) was suspended in a 20 mM phosphate buffer (pH 5.5). The starch suspension was heated in a water-bath thermostatic shaker for 10 min at 55 °C. The starch paste was modified with BA (50 U/g dry-weight starch) and stirred at 600 r/min for 2 h at 55 °C. Then, different amounts of GT (3000, 5000, 7000, and 9000 U/g) were added and stirred for another 6 h at 600 r/min. Immediately after the reaction, the solution was heated at 100 °C for 10 min to inactivate the enzyme and centrifuged (4000 r/min, 10 min), and distilled water was rinsed repeatedly, and the upper lipid layer and impurities were scraped off. The enzyme-modified maize starch sample was dried in an oven at 40 °C for 24 h and passed through a 100-mesh sieve [15].

#### 2.2.2. Determination of Amylose and Amylopectin Ratio

This was determined using the amylose assay kit (K-AMYL, Megazyme Reagents Ireland, Bray, Ireland). Precisely, a starch sample of 20 mg was weighed into a 10 mL centrifuge tube. Add 1 mL of dimethyl sulfoxide to the centrifuge tube, heat for 15 min, and vortex. Add 6 mL of 95% (*v*/*v*) ethanol and vortex. Leave to stand for 15 min. Centrifuge, discard the supernatant, and use the precipitate for subsequent amylose and amylopectin determinations. Add 2 mL of DMSO to the starch pellet and place the centrifuge tube in a boiling water bath for 15 min with occasional stirring. Immediately after removing the tube from the boiling water bath, 4 mL of Con A solvent was added and mixed thoroughly, and the Con A solvent was diluted to volume in a 25 mL volumetric flask. Take 1.0 mL of the above solution. According to the principle of ConA precipitation of amylose, the absorbance of Con A supernatant was measured at 510 nm. Mix 0.5 mL of the above solution with 4 mL of 100 mM sodium acetate buffer (pH 4.5). Add 0.1 mL of mixed enzyme solution and incubate at 40 °C for 10 min. Transfer 1.0 mL of the solution to a glass test tube, add 4 mL of GOPOD reagent, and mix well. Incubate at 40 °C for 20 min. Measure the absorbance of total starch aliquot of the sample at 510 nm. The content of amylose and amylopectin was then calculated [22].

#### 2.2.3. Determination of Starch Granule Morphology

The starch samples were uniformly dispersed on the surface of the double-sided carbon tape and sprayed with a layer of gold–palladium. The microstructure of the maize starch samples before and after the dual enzyme modification was observed using a scanning electron microscope (MERLIN Compact, Carl Zeiss Inc., Jena, Germany) at 5 KV, 3.5 spot size, 30 µm objective aperture, 7.4 mm working distance, and 1000× magnification [23].

#### 2.2.4. Determination of Starch Crystalline Properties

The crystal structure of starch samples before and after dual enzyme modification was analyzed by X-ray diffraction (D/MAX2500, Rigaku Corporation, Akishima, Tokyo, Japan). Referring to the study of Gu et al. [24], the samples were scanned in 0.02° steps over the range of 4°–40° (2θ) at a rate of 4°/min. The relative crystallinity of each starch sample was calculated using MDI Jade 6.0 software.

#### 2.2.5. Determination of Fourier Transforms Infrared (FTIR) Spectroscopy

The FT–IR spectra of starch samples before and after dual enzyme modification were determined using an FT–IR spectrophotometer (Bruker Company, Karlsruhe, Germany). The scanning wavelength range was 4000~400 cm^−1^. The resolution was 4 cm^−1^, and the number of scans was 64 [25]. The ground samples were mixed with dried KBr (1:100 *w*/*w*) and pressed into flakes. The spectrum was deconvoluted by OMNIC 8.2 software. The ratio (1047 cm^−1^/1022 cm^−1^) of the intensity was calculated from the deconvoluted spectra.

#### 2.2.6. Determination of Starch Branching Degree

The Proton Nuclear Magnetic Resonance (^1^H NMR) was carried out using proton nuclear magnetic resonance spectroscopy (Avance III-400 MHz, Bruker Co., Saarbrücken, Germany). The scanning frequency of the instrument used was 32, the resonance radio frequency was 500.23 MHz, and the nuclear magnetic spectrum was ^1^H. The supernatant was removed and added to a magnetic resonance tube for machine detection. a-1,4 and a-1,6 linkages were quantified with panose as a standard [26]. The data was analyzed using MestR-Nova V 14.2 software, and the results were obtained by selecting the peak range based on the sample peak time. Calculation was by Equation (1):

(1) $$\mathrm{DB}\left(\%\right)=A/(A+B)\times 100\%$$

DB: branching degree

*A*: peak area of the α-1,6 bond

*B*: Peak area of α-1,4 bond [27]

#### 2.2.7. Determination of Starch Chain-Length Distribution

Starch chain-length distribution was determined using the methods of Ren et al. [26] and Chung et al. [28] with appropriate modifications. The chain length distribution of debranched starch samples was determined using high-performance anion exchange chromatography (ICS-5000, Thermo Fisher Scientific Co., Ltd., Waltham, MA, USA). A total of 10.0 mg of starch sample was mixed with 4 mL of 90% DMSO solution and stirred in a boiling water bath for 20 min, and 6 mL of anhydrous ethanol was added and centrifuged at 3000 rad/min for 15 min. The supernatant was discarded. Next, 2 mL of 50 mM sodium acetate buffer (pH 4.0) was added and balanced in a water bath for 20 min at 40 °C for 10 min. Add 10 μL of isoamylase, 40 °C, 150rad/min, leave for 24 h, then boil for 10 min to inactivate the enzyme. Sample 1mL of the solution, dilute to 5 mL with 150 mM NaOH, filter the membrane, and inject into the machine. Samples were measured and analyzed at 35 °C using an eluent gradient and a flow rate of 0.5 mL/min. 250 mM sodium hydroxide (eluent A), 1 M sodium acetate (eluent B), and ultrapure water (eluent C). A CarboPac PA200 column (3 × 200 mm, Thermo Fisher Scientific Co., Ltd., Waltham, MA, USA) was used.

#### 2.2.8. Determination of Starch Thermal Properties

The thermal properties of starch samples before and after dual enzyme modification were analyzed by DSC (Discovery SDT650, TA Instruments, New Castle, DE, USA) [29]. The aluminum pan containing 3 mg (dry base) of sample and 9 mL of water was sealed and put at 4 °C for 24 h before analysis [30,31]. This was ramped up to 120 °C at 10 °C/min.

#### 2.2.9. Determination of Starch Pasting Properties by RVA

The adhesion properties of the samples were measured using a Rapid Visco analyzer (RVA) (model 3D+, Newport Scientific, Warriewood, Australia). Starch samples (3 g, dry basis, db) were suspended in 25 mL of distilled water. The samples were equilibrated at 50 °C for 1 min, then heated to 95 °C at 12 °C/min and held at 95 °C for 5 min. The hot sample was then cooled to 50 °C at 12 °C/min and held at 50 °C for 4 min. Paddle speed was 160 rpm [32].

#### 2.2.10. Determination of Starch Light Transmittance

The starch sample (10 g) was taken with deionized water to prepare a starch emulsion with a mass concentration of 1%. The starch paste was continuously heated and stirred in a boiling water bath for 30 min until the starch paste was cooled down to 25 °C, and the transmittance of the starch paste at 640 nm was determined using the microplate reader (Spectramax 190, Molecular Devices, Inc., Silicon Valley, NC, USA) with distilled water as a reference [33].

#### 2.2.11. Determination of Starch Solubility and Swelling Power

Weigh a certain amount of maize starch samples (dry starch basis) and prepare a 50 mL starch suspension with a concentration of 6%. The starch suspension was stirred, heated at 80 °C for 30 min and then cooled to room temperature. The starch suspension was centrifuged for 20 min at 4000 r/min to separate the supernatant and precipitate. The supernatant was collected, dried to a constant weight, and weighed. Solubility (S) and swelling power (SP) were calculated as in Equations (2) and (3) [34]:

(2) $$S\left(\%\right)=A/W\times 100$$

(3) $$SP\left(\%\right)=B/W(1-S)\times 100$$

*A*: the mass of the supernatant after drying to transverse weight (g)

*W*: the quality of the dry starch sample, (g)

*B*: the quality of sediment after centrifugation, (g)

#### 2.2.12. Statistical Analysis

All experimental data in this study are expressed as standard deviation ± mean. All experiments were performed in triplicate. Statistical analysis was performed using SPSS using one-way analysis of variance (ANOVA) with Duncan multiple comparison test. Plotting was done using Origin 2018 software. Correlation analyses among different parameters were determined with Spearman correlation coefficients. Differences resulting in values of *p* < 0.05 were considered significant.

## 3. Results and Discussion

### 3.1. Effect of Dual Enzyme Modification on the Ratio of Amylose/Amylopectin

The effect of dual enzyme modification on the ratio of amylose /amylopectin in maize starch is shown in Table 1. The amylose starch content of natural maize starch was 28.77%. The amylose starch content of maize starch samples after β-amylase modification was reduced to 26.16%. The amylose starch content of the bi-enzymatic-modified samples showed a decreasing tendency with the increase in the addition of α-glucosidase, implying the conversion of amylose to branched molecule [15]. This is because amylose starch mainly consists of α-1,4-glycosidic linkages and amylopectin starch consists of α-1,4-glycosidic linkages and α-1,6-glycosidic linkages [13]. β-Amylase hydrolyzes α-1,4-glycosidic linkages step by step along the non-reducing end and stops hydrolyzing when it encounters the branching point, so its degree of enzymatic hydrolysis is limited. The dual enzyme modification doubly hydrolyzes the α-1,4-glycosidic bond and increases the branching degree in the form of the α-1,6-glycosidic bond so that the relative content of amylose decreases [35]. Ren et al. [26] improved the conversion of amylose to amylopectin using a two-stage modification of 1,4-α-glucan branching enzyme.

### 3.2. Effect of Dual Enzyme Modification on Maize Grain Morphology

**Figure 1:**
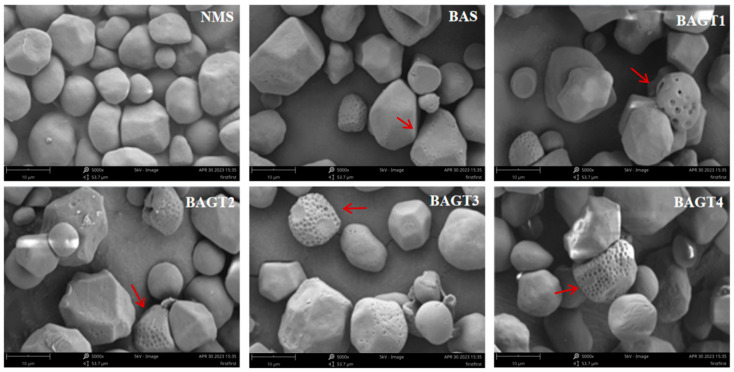
SEM of native maize starch (NMS); enzyme-modified starches of BA (BAS) and enzyme-modified starches of BA + GT (different amounts): (BAGT1) 3000 U/g; (BAGT2) 5000 U/g; (BAGT3) 7000 U/g; (BAGT4) 9000 U/g.

Scanning electron micrographs of the maize starch samples before and after dual enzyme modification are shown in Figure 1. The natural maize starch granules were ellipsoidal or irregular polyhedral, with relatively smooth granule surfaces, intact granules, and only a few micropores [36]. The granule morphology of the bis-enzyme-modified maize starch was significantly changed. More pores of smaller size were formed on the surface of BAS granules compared to natural maize starch. The number of pores on the surface of starch granules increased, and their size was larger after dual enzyme modification; the pores’ depth that was formed gradually penetrated the granules’ internal center. Zhong et al. [37] modified maize starch with malt α-amylase, resulting in the formation of extensive pores in the starch granules. With the increase in α-glucosidase addition, a small portion of starch granules were fragmented, which might be caused by excessive enzymatic hydrolysis [38]; it can also be seen from the changes in the morphology of the granules that the effect of dual enzyme-modified starch granules is stronger than the effect of a single enzyme.

### 3.3. Effect of Dual Enzyme Modification on the Crystal Structure of Maize Starch

**Figure 2:**
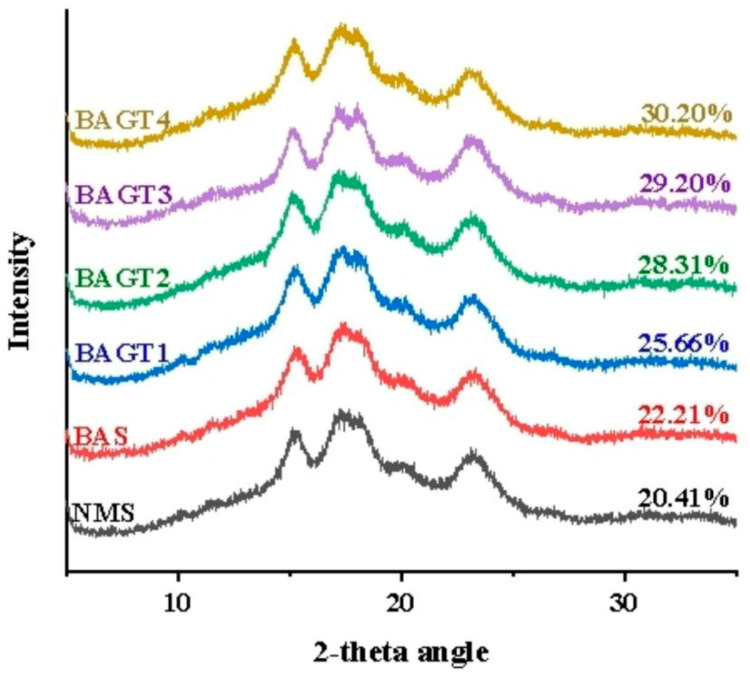
X-ray diffraction pattern analysis of native maize starch (NMS), enzyme-modified starches of BA (BAS) and enzyme-modified starches of BA + GT (different amounts): (BAGT1) 3000 U/g; (BAGT2) 5000 U/g; (BAGT3) 7000 U/g; (BAGT4) 9000 U/g.

Figure 2 and Table 2 show the XRD patterns and relative crystallinity of native and enzyme-modified starch. Natural maize starch showed major firm diffraction peaks near 2θ = 15°, 17°, 18°, and 23°, indicating a clear A-type crystal structure [39]. Figure 2 shows that enzymatic hydrolysis did not alter the XRD profile of the starch, indicating that the addition of the enzyme did not disrupt the crystalline regions. The relative crystallinity of natural maize starch was 20.41%. The crystallinity of the starch samples after dual enzyme modification increased gradually with the increase of α-glucosidase. In the starch granules, a small portion of amylose starch and amylopectin starch had many branches, combining to form a compact crystal structure; the crystalline region was highly ordered, while the size of the relative content of amylose starch determined the size of the amorphous region [40]. The content of amylopectin was positively correlated with relative crystallinity (R = 0.993, *p* < 0.01) [41]. As the dual enzyme modification decreases the content of amylose in maize starch granules, the action of β-amylase and α-glucosidase on the starch results in the addition of more amylopectin, which entangles the starch more tightly and leads to a higher relative crystallinity. In the study by Jo et al. [42], starch was modified by glycogen branching enzyme and amylo-sucrase. Increased relative crystallinity, branch density, and chain length distribution of amylopectin were altered.

### 3.4. Effect of Enzymatic Modifications on the Short-Range Ordered Structure of Maize Starch

**Figure 3:**
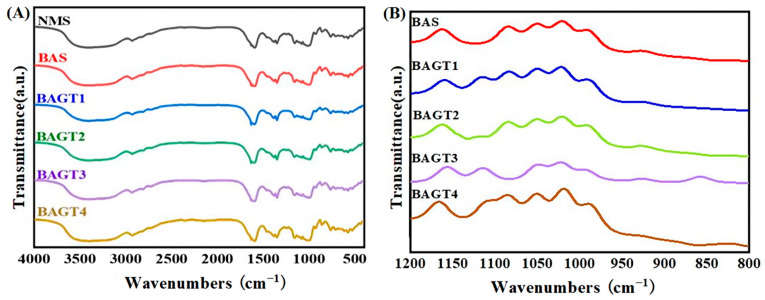
FTI–R spectra (**A**) and deconvoluted FT-IR spectra (**B**) of native maize starch (NMS); enzyme-modified starches of BA (BAS) and enzyme-modified starches of BA + GT (different amounts): (BAGT1) 3000 U/g; (BAGT2) 5000 U/g; (BAGT3) 7000 U/g; (BAGT4) 9000 U/g.

FT–IR spectra of native maize starch and enzyme-modified starch are shown in Figure 3. The infrared spectra of dual enzyme-modified maize starch showed no change in the peak shape from that of the natural maize starch, except for a slight difference in the peaks corresponding to the peak tips. This indicates that no new functional groups were introduced into the dual enzyme-modified maize starch compared to natural examples. According to Chen et al. [43], the sensitive region of infrared spectra of starch molecular structure is in the range of 1200 cm^−1^~800 cm^−1^. In FT–IR spectra, the IR absorption band of starch at 1047 cm^−1^ is related to the structural features of the crystalline region and the IR absorption band at 1022 cm^−1^ is related to the structural features of the amorphous region [44]. Thus, the 1047 cm^−1^ and 1022 cm^−1^ ratios indicate the amount of ordered crystalline over amorphous areas of starch. The ratio can reflect the short-range ordered structure of starch, and a decrease in the ratio suggests that the starch is moving from ordered to disordered [45]. From Table 2, compared with natural maize starch, the R1047 cm^−1^/1022 cm^−1^ values of the samples increased with the deepening of the enzymatic digestion of the double enzyme-modified starch. This may be because the amylose/amylopectin ratio of the enzyme-modified starch samples gradually decreased, and the amorphous zone is mainly composed of amylose starch; the decrease in the content of amylose starch indicates that the relative crystallinity of the starch increases, the degree of order increases, and the R-value rises, and the result agrees with the results of XRD.

### 3.5. Effect of Dual Enzyme Modification on the Degree of Branching of Maize Starch

**Figure 4:**
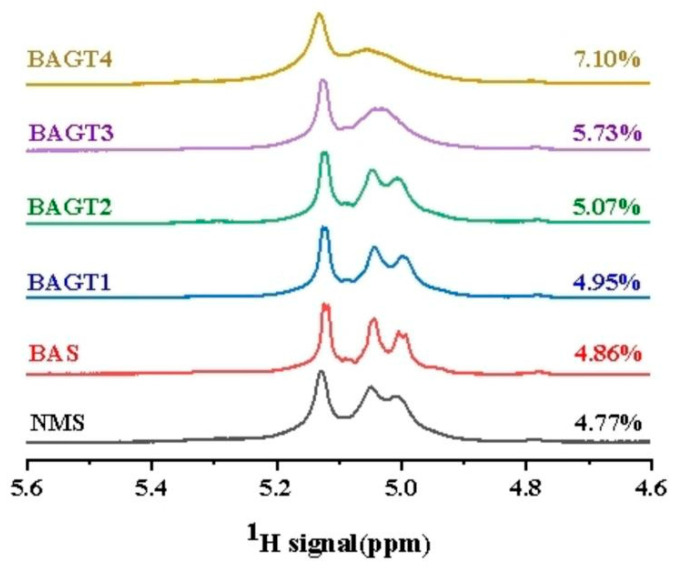
^1^HNMR analysis of native maize starch (NMS), enzyme-modified starches of BA (BAS) and enzyme-modified starches of BA + GT (different amounts): (BAGT1) 3000 U/g; (BAGT2) 5000 U/g; (BAGT3) 7000 U/g; (BAGT4) 9000 U/g.

The branching degree of starch before and after dual enzyme modification can be analyzed based on the starch α-1,6: α-1,4 chain ratio, as shown in Figure 4, and the ^1^HNMR spectrum shows four starch peaks in the region of 4.6~5.6 ppm. Similar to the results of Zhong et al. [27], the chemical shift of α-1,4 was 5.12 ppm, and that of α-1,6 was 4.78 ppm [46]. The branching degree of the dual enzyme-modified starch increased gradually compared with that of the natural maize starch. β-amylase alone and α-glucosidase modification of starch increased the relative ratios of the α-1,6 linkages to the α-1,4 linkages. The increase in the branching degree was more pronounced for β-amylase and α-glucosidase-modified starch, which were 4.95%, 5.07%, 5.73%, and 7.10%, respectively. The higher the dosage of α-glucosidase, the higher the ratio. The results showed that β-amylase increased the branching degree by hydrolyzing the α-1,4 linkages of starch, but with limited effect. α-Glucosidase not only severed the α-1,4 linkages but also transferred free glucose residues in the form of α-1,6-glucosidic linkages to the same or different amylose and amylopectin chains and promoted the formation of new branching points [37]. Dual enzyme modification increases the branching degree of starch by catalyzing hydrolysis and transfer reactions to form new l, 6 glycosidic linkages [42]. Li et al. [47] used a combination of amylopectin, β-amylase and trans-glucosidase to modify sweet potato starch in a sequential manner, with the degree of branching gradually increasing from 24% to 32%.

### 3.6. Effect of Dual Enzyme Modification on the Chain-Length Distribution of Maize Starch

The effect of dual enzyme modification on the chain length distribution of maize starch is shown in Table 3. It has been reported [48] that the chain-length distribution was divided into four parts based on the degree of polymerization (DP) of the chain-lengths: A (DP ≤ 12), B1 (DP 13–24), B2 (DP 25–36), and B3 (DP ≥ 37) [49,50]. As the amylose/amylopectin ratio decreased from 1:3 to 1:4.5, the proportion of A and B1 chains increased from 14.97% and 24.92% to 17.87% and 27.44%, respectively, while the proportion of B2 and B3 chains decreased from 12.26 and 47.86% to 10.48% and 44.21%, respectively. The increase in A chains may be due to the action of enzymes on starch molecules to hydrolyze the long chains into shorter ones [51]. At the same time, β-amylase hydrolyzed the α-1,4-glycosidic bond, and α-glucosidase attached the hydrolyzed glucosides bond to amylose starch in the form of α-1,6-glycosidic bond, increasing the A chain. The increase in the B1 chain may be due to the hydrolyzed glycosidic linkages attached to amylopectin by α-glucosidase after the action of the dual enzyme on the starch molecule; the branched chains of amylopectin and side chains with shorter outer chains increased after the action, and the branching degree of starch increased, which is consistent with the results of starch branching degree. The decrease of B2 and B3 chains may be due to the decomposition of long side chains and outer chains after the enzyme action on starch molecules [52], the average chain-length of the starch samples decreased due to the hydrolysis combined with the trans-glycosylation, and the average chain-length of the starch samples decreased. β-Amylase and α-glucosidase hydrolyzed the longer side chains, the outer chains, and the shorter chains of amylopectin starch of the starch granules [53]. Treatment of granular maize starch with 1,4-α glucan branching enzyme occurs in the study of Ren et al. [26]. As the 1,4-alpha glucan branching enzyme tends to act on longer branches, the proportion of A and B2 chains in starch increases.

### 3.7. Effect of Dual Enzyme Modification on the Thermal Properties of Maize Starch

Differential scanning calorimetry was used to analyze the thermal properties of NMS, BAS (β-amylase modified maize starch), and BAGTS (β-amylase and α-glucosidase modified maize starch). The results are shown in Table 4. The pasting temperature is closely related to the crystal structure [54], granule size, and amylose content of starch granules [55]. In general, the lower the amylose content, the lower the pasting temperature [56]. Lin et al. [57] showed that the pasting temperature was positively correlated with the longer branch-chain of amylopectin and negatively correlated with the short branch-chain of amylopectin. Due to the dual enzyme modification, the content of maize starch amylose is reduced, the longer branch-chain content is reduced, and its pasting temperature is reduced; however, due to the limited degree of enzymolysis, the starch granules were not destroyed, and the trend in the paste temperature change was less pronounced. The enthalpy of pasting (ΔH) reflects the amount of unraveling of the double helix during pasting and represents the disordered nature of the double helix [58]. As shown in Table 4, the enthalpy of pasting of double enzyme-modified amylose showed a decreasing trend; with the increase in enzyme addition, the number of long chains decreased, the proportion of short chains increased, the content of amylose decreased, and the content of amylose that needs to open the double-helix structure decreased, which in turn decreased the ΔH.

### 3.8. Effect of Dual Enzyme Modification on the Pasting Property Parameters of Maize Starch

When the starch solution reaches a specific temperature, the pasting phenomenon will occur, the hydrogen bond between starch molecules will be broken, the amylose will be precipitated, and the viscosity of starch emulsion will be increased until the formation of gel, and the pasting characteristic of starch is one of the essential indexes reflecting the quality of starch [54]. In RVA, maize starch and water are mixed in a machine, causing a series of changes in viscosity through heating and cooling [59]. As the temperature rises, the starch granules in the suspension swell dramatically, the viscosity increases rapidly, and the suspension gradually enters a gel state when it reaches the peak, known as the peak viscosity [60]. The peak viscosity of the dual enzyme-modified starch was significantly higher than that of natural starch. The peak viscosity of the dual enzyme-modified starch samples showed a decreasing trend with the increase in enzyme addition, possibly because the peak viscosity is related to the degree of completion of the starch granules. With the increase in enzyme activity, the starch granules were hydrolyzed by the enzyme to a higher degree, which weakened the granular structure of starch, and the starch could not expand to the original maximum value.

The disintegration value reflects the shear resistance of starch granules and is the difference between peak viscosity. Higher disintegration values represent the starch’s resistance to shear during swelling, reflecting the stability of starch granules. The BD of the double enzyme-modified starch samples showed a decreasing trend with the deepening of the enzymatic degradation [61]. Table 5 shows that the BD of the natural maize starch granules was lower than that of the double enzyme-modified starch. There is a correlation between the size of the BD and the PV, with a higher PV indicating that the starch granules swelled more and thus decomposed more, resulting in a more significant BD value [62].

Regrowth value is an essential index for evaluating the difficulty of starch regrowth. Starch milk regrowth or rearrangement occurs after the temperature gradually decreases. The lower the regrowth value, the stronger the starch resistance to regrowth, representing better starch resistance to regrowth. The regrowth value of BAGTS was lower compared to that of NMS and BAS. The rapid recrystallization of amylose is mainly responsible for starch’s short-term regrowth, and starch with a high content of amylose is more prone to regrowth. As the degree of enzymolysis deepens, the content of amylose starch decreases, and the regrowth value also decreases.

### 3.9. Effect of Dual Enzyme Modification on the Light Transmittance of Maize Starch

When light passes through this starch suspension, light penetration, refraction, and reflection occur. Starch transmittance is an important quality parameter in starch food processing [63]. The transmittance of starch decreases with the increase in the amylose content. Due to the combination of amylose starch molecules to form larger granules or bundles, when the volume increases to a certain extent, the starch forms coagulation, and light is not easy to pass through. As shown in Table 6, the transmittance of the enzyme-modified starch samples improved compared to natural maize starch. With the increase in α-glucosidase addition in BAGTS, the transmittance of starch increased. This may be because enzyme-modified starch has a lower amylose content and a lower transmittance.

### 3.10. Effect of Dual Enzyme Modification on Solubility and Swelling Capacity of Maize Starch

The effect of dual enzyme modification on the solubility and swelling of maize starch is shown in Table 6, where the solubility of natural maize starch is 20.15% and the swelling is 33.97%. With the increase in enzyme addition, the solubility of starch decreased, and the swelling degree increased, because the solubilization of starch mainly caused the escape of amylose molecules from the swollen starch granules [64]. Thus, the lower the amylose content of starch, the fewer amylose molecules escape and the lower the solubility. The swelling of starch is due to the characteristics of amylopectin starch, such as double helix structure and chain-length distribution; thus, the higher the content of amylopectin in starch granules, the higher the swelling degree. Han et al. [65] modified maize starch with α-amylase and saccharolytic enzymes, reducing solubility and increasing the swelling properties of maize starch.

## 4. Conclusions

In this study, dual-modification of maize starch was carried out using β-amylase and α-Glucosidase. The results showed that the dual-enzyme treatment changed the amylose/amylopectin ratio of maize starch, and the reduction of amylose was evident with the increase of α-glucosidase addition, which was attributed to the gradual hydrolysis of the α-1,4-glycosidic bond by the β-amylase enzyme along the non-reducing end, which was limited in its degree of enzymolysis. α-Glucosidase not only hydrolyzed the α-1,4-glycosidic bond but also increased the degree of branching in the form of an α-1,6-glycosidic bond so that the content of amylose was decreased. Moreover, as the amylose/amylopectin ratio changed, the granular morphology of the enzyme-treated starch samples changed to different degrees. Since the amylose/amylopectin ratio was related to the composition of amorphous regions, the relative crystallinity and short-range ordering degree of starch increased with the decrease in amylose content after double enzyme treatment. With the deepening of enzymolysis, the branching degree of starch molecules increased, the long chains decreased, and the short chains increased; β-amylase and α-glucosidase acted together on the side-chain outer chains and shorter amylopectin of maize starch. The enthalpy of pasting of starch decreased with the deepening of the enzymolysis, the aging resistance increased, and the transmittance of starch increased due to the reduction of amylose, increasing the swelling rate of starch and decreasing the solubility of starch. The starch sample of BAGT2 has higher shear resistance and lower solubility, which is suitable for applications such as edible films and heat-sensitive materials. The starch sample of BAGT4 has lower viscosity, which is ideal for applications such as adhesives and thickeners. This study provides valuable ideas and references for developing and applying enzyme-modified starch technology and preparing functional starch. In future work, there is a need to investigate further the role of bi-enzymatic co-modification on the fine structure and physicochemical function of starch and continue to develop methods for complex modification of starch.

## Figures and Tables

**Table 1:** Content of amylose and amylose /amylopectin ratio in maize starch samples (based on dry starch) before and after dual enzyme modification.

Sample	Amylose % (*w*/*w*)	Amylopectin% (*w*/*w*)	Amylose/Amylopectin
NMS	28.77 ± 0.86 ^a^	70.79 ± 0.10 ^f^	1: 2.42 ± 0.10 ^e^
BAS	26.16 ± 0.37 ^b^	73.27 ± 0.13 ^e^	1: 2.74 ± 0.05 ^d^
BAGT1	25.16 ± 0.69 ^c^	74.48 ± 0.03 ^d^	1: 2.92 ± 0.11 ^cd^
BAGT2	23.88 ± 0.21 ^c^	75.35 ± 0.02 ^c^	1: 3.05 ± 0.04 ^c^
BAGT3	22.28 ± 0.67 ^d^	77.17 ± 0.00 ^b^	1: 3.38 ± 0.28 ^b^
BAGT4	18.60 ± 0.99 ^e^	81.71 ± 0.05 ^a^	1: 4.47 ± 0.28 ^a^

Abbreviations: NMS: native maize starch; BAS: enzyme-modified starches of BA; BAGT1: enzyme-modified starches of BA + (3000 U/g) GT; BAGT2: enzyme-modified starches of BA and (5000 U/g) GT; BAGT3: enzyme-modified starches of BA and (7000 U/g) GT; BAGT4: enzyme-modified starches of BA and (9000 U/g) GT. The results are the means ± standard deviations (*n* = 3). Different letters after each column of data show significance (*p* < 0.05).

**Table 2:** Relative crystallinity and R_1047 cm_^−1^_/1022 cm_^−1^ of maize starch before and after dual enzyme modification.

Samples	Relative Crystallinity (%)	R_1047 cm_^−1^_/1022 cm_^−1^
NMS	20.41	0.75 ± 0.02 ^d^
BAS	22.21	0.77 ± 0.00 ^d^
BAGT1	25.66	0.78 ± 0.00 ^d^
BAGT2	28.31	0.87 ± 0.03 ^c^
BAGT3	29.20	0.93 ± 0.01 ^b^
BAGT4	30.20	1.19 ± 0.05 ^a^

Abbreviations: NMS: native maize starch; BAS: enzyme-modified starches of BA; BAGT1: enzyme-modified starches of BA + (3000 U/g) GT; BAGT2: enzyme-modified starches of BA and (5000 U/g) GT; BAGT3: enzyme-modified starches of BA and (7000 U/g) GT; BAGT4: enzyme-modified starches of BA and (9000 U/g) GT. The results are the means ± standard deviations (*n* = 3). Different letters after each column of data show significance (*p* < 0.05).

**Table 3:** Chain-length distribution of maize starch before and after enzymatic modification.

Sample	Chain-Length Distribution Ratio (%)			
	A (DP3–12)	B1 (DP13–24)	B2 (DP25–36)	B3 (DP ≥ 37)
NMS	14.97	24.92	12.26	47.86
BAS	15.15	25.81	11.74	47.30
BAGT1	16.16	25.73	11.18	46.93
BAGT2	16.19	26.51	11.27	46.03
BAGT3	16.21	26.66	11.16	45.79
BAGT4	17.87	27.44	10.48	44.21

Abbreviations: NMS: native maize starch; BAS: enzyme-modified starches of BA; BAGT1: enzyme-modified starches of BA + (3000 U/g) GT; BAGT2: enzyme-modified starches of BA and (5000 U/g) GT; BAGT3: enzyme-modified starches of BA and (7000 U/g) GT; BAGT4: enzyme-modified starches of BA and (9000 U/g) GT. The results are the means ± standard deviations (*n* = 3). Different letters after each column of data show significance (*p* < 0.05).

**Table 4:** Thermodynamic parameters of maize starch samples (based on dry starch) before and after dual enzyme modification.

Sample	T_o_ (°C)	T_p_ (°C)	T_c_ (°C)	ΔH (J/g)
NMS	48.26 ± 15.43 ^b^	70.09 ± 0.17 ^a^	95.07 ± 14.98 ^a^	13.68 ± 4.93 ^a^
BAS	55.30 ± 8.03 ^ab^	70.56 ± 0.00 ^d^	87.68 ± 13.50 ^ab^	11.35 ± 0.79 ^a^
BAGT1	67.15 ± 0.51 ^a^	71.08 ± 0.00 ^a^	77.27 ± 0.31 ^b^	10.94 ± 0.41 ^a^
BAGT2	64.00 ± 1.03 ^a^	70.54 ± 0.02 ^d^	79.39 ± 2.17 ^ab^	10.66 ± 1.20 ^a^
BAGT3	66.63 ± 0.21 ^a^	70.79 ± 0.00 ^c^	78.52 ± 0.39 ^ab^	10.21 ± 0.33 ^a^
BAGT4	66.53 ± 0.31 ^a^	70.87 ± 0.00 ^b^	76.14 ± 0.37 ^b^	9.13 ± 0.30 ^a^

Abbreviations: NMS: native maize starch; BAS: enzyme-modified starches of BA; BAGT1: enzyme-modified starches of BA + (3000 U/g) GT; BAGT2: enzyme-modified starches of BA and (5000 U/g) GT; BAGT3: enzyme-modified starches of BA and (7000 U/g) GT; BAGT4: enzyme-modified starches of BA and (9000 U/g) GT. The results are the means ± standard deviations (*n* = 3). Different letters after each column of data show significance (*p* < 0.05).

**Table 5:** The pasting properties parameters of maize starch samples (based on dry starch) before and after dual enzyme modification.

Sample	PV (cp)	TV (cp)	BD (cp)	FV (cp)	SB (cp)
NMS	2720.00 ± 20.66 ^d^	1662.67 ± 14.50 ^c^	1058.67 ± 34.93 ^c^	2460.00 ± 20.31 ^d^	798.00 ± 13.98 ^d^
BAS	3975.67 ± 47.37 ^a^	2170.00 ± 28.09 ^a^	1805.00 ± 20.71 ^b^	3783.50 ± 38.59 ^a^	1613.67 ± 20.50 ^a^
BAGT1	3861.67 ± 40.61 ^b^	1807.50 ± 19.20 ^b^	2054.50 ± 14.12 ^a^	3371.50 ± 40.35 ^b^	1564.00 ± 38.09 ^a^
BAGT2	3841.00 ± 18.89 ^b^	1845.00 ± 12.83 ^b^	1996.50 ± 27.07 ^a^	3294.00 ± 34.24 ^b^	1449.00 ± 24.14 ^b^
BAGT3	3674.33 ± 45.96 ^c^	1743.50 ± 18.28 ^b^	1930.00 ± 52.32 ^a^	3175.33 ± 36.97 ^c^	1432.50 ± 16.97 ^b^
BAGT4	3578.00 ± 46.67 ^c^	1558.50 ± 19.90 ^c^	2020.00 ± 33.23 ^a^	2020.00 ± 23.93 ^e^	1281.00 ± 15.97 ^c^

Abbreviations: Where PV indicates peak viscosity; TV indicates through viscosity; FV indicates final viscosity; BD indicates breakdown viscosity; SB, setback viscosity; NMS: native maize starch; BAS: enzyme-modified starches of BA; BAGT1: enzyme-modified starches of BA + (3000 U/g) GT; BAGT2: enzyme-modified starches of BA and (5000 U/g) GT; BAGT3: enzyme-modified starches of BA and (7000 U/g) GT; BAGT4: enzyme-modified starches of BA and (9000 U/g) GT. The results are the means ± standard deviations (*n* = 3). Different letters after each column of data show significance (*p* < 0.05).

**Table 6:** Transmittance, solubility, and swelling power rate of maize starch samples (based on dry starch) before and after dual enzyme modification.

Sample	Transmittance (%)	Solubility (%)	Swelling Power (%)
NMS	1.40 ± 0.43 ^d^	20.15 ± 0.15 ^a^	33.97 ± 0.25 ^f^
BAS	1.42 ± 0.02 ^d^	17.03 ± 0.17 ^b^	39.47 ± 0.12 ^e^
BAGT1	1.61 ± 0.14 ^c^	15.72 ± 0.23 ^c^	41.56 ± 0.33 ^d^
BAGT2	1.93 ± 0.08 ^b^	15.64 ± 0.07 ^c^	42.76 ± 0.18 ^c^
BAGT3	2.04 ± 0.03 ^a^	14.20 ± 0.13 ^d^	44.40 ± 0.13 ^b^
BAGT4	2.16 ± 0.05 ^a^	13.76 ± 0.07 ^e^	45.79 ± 0.07 ^a^

Abbreviations: NMS: native maize starch; BAS: enzyme-modified starches of BA; BAGT1: enzyme-modified starches of BA + (3000 U/g) GT; BAGT2: enzyme-modified starches of BA and (5000 U/g) GT; BAGT3: enzyme-modified starches of BA and (7000 U/g) GT; BAGT4: enzyme-modified starches of BA and (9000 U/g) GT. The results are the means ± standard deviations (*n* = 3). Different letters after each column of data show significance (*p* < 0.05).

## Data Availability

The original contributions presented in this study are included in the article. Further inquiries can be directed to the corresponding author.

## References

[1] Kaur B., Ariffin F., Bhat R., Karim A.A. (2012). Progress in starch modification in the last decade. Food Hydrocoll..

[2] Lin D., Zhao J., Wang Z., Qin W., Wu Z. (2023). Effects of glutathione on structural and digestibility properties of high-hydrostatic-pressure-gelatinized maize starch with different amylose/amylopectin ratios. LWT.

[3] Yang Z., Swedlund P., Hemar Y., Mo G., Wei Y., Li Z., Wu Z. (2016). Effect of high hydrostatic pressure on the supramolecular structure of corn starch with different amylose contents. Int. J. Biol. Macromol..

[4] Li J., Kong X., Ai Y. (2022). Modification of granular waxy, normal and high-amylose maize starches by maltogenic α-amylase to improve functionality. Carbohydr. Polym..

[5] Han S., Hu Y., Li C., Yu Y., Wang Y., Gu Z., Hao Z., Xiao Y., Liu Y., Liu K. (2024). Exploring the formation mechanism of resistant starch (RS3) prepared from high amylose maize starch by hydrothermal-alkali combined with ultrasonic treatment. Int. J. Biol. Macromol..

[6] Cahyana Y., Wijaya E., Halimah T.S., Marta H., Suryadi E., Kurniati D. (2019). The effect of different thermal modifications on slowly digestible starch and physicochemical properties of green banana flour (*Musa acuminata colla*). Food Chem..

[7] Milani J.M., Golkar A., Galanakis C.M. (2021). Chapter 8—Starch modification by novel technologies and their functionality. Food Structure and Functionality.

[8] Cahyana Y., Putri Y.S.E., Solihah D.S., Lutfi F.S., Alqurashi R.M., Marta H. (2022). Pickering Emulsions as Vehicles for Bioactive Compounds from Essential Oils. Molecules.

[9] Handarini K., Hamdani J.S., Cahyana Y., Setiasih I.S. (2020). Gaseous Ozonation at Low Concentration Modifies Functional, Pasting, and Thermal Properties of Arrowroot Starch (*Maranta arundinaceae*). Starch-Starke.

[10] Zhang J., Ran C., Jiang X., Dou J. (2021). Impact of octenyl succinic anhydride (OSA) esterification on microstructure and physicochemical properties of sorghum starch. LWT.

[11] Xu Y., Wu Y.-j., Sun P.-l., Zhang F.-m., Linhardt R.J., Zhang A.-q. (2019). Chemically modified polysaccharides: Synthesis, characterization, structure activity relationships of action. Int. J. Biol. Macromol..

[12] Cahyana Y., Annisa N.D.N., Khoerunnisa T.K., Sulastri S., Marta H., Rialita T., Yuliana T., Aït-Kaddour A., Şumnu G. (2024). Banana starch modified by heat moisture treatment and annealing: Study on digestion kinetics and enzyme affinity. Int. J. Biol. Macromol..

[13] Qiu Z., Li R., Chen J., Chen L., Xie F. (2024). Favored CH-π interaction between enzymatically modified high amylose starch and resveratrol improves digestion resistance. Food Hydrocoll..

[14] Shah A., Masoodi F.A., Gani A., Ashwar B. (2018). Dual enzyme modified oat starch: Structural characterisation, rheological properties, and digestibility in simulated GI tract. Int. J. Biol. Macromol..

[15] Li Y., Li C., Gu Z., Cheng L., Hong Y., Li Z. (2019). Digestion properties of corn starch modified by α-D-glucan branching enzyme and cyclodextrin glycosyltransferase. Food Hydrocoll..

[16] Karakelle B., Kian-Pour N., Toker O.S., Palabiyik I. (2020). Effect of process conditions and amylose/amylopectin ratio on the pasting behavior of maize starch: A modeling approach. J. Cereal Sci..

[17] Yuan M., Wang Y., Bai Y., Svensson B. (2022). Distinct effects of different α-amylases on cross-linked tapioca starch and gel-improving mechanism. Food Hydrocoll..

[18] Ji H., Li X., Bai Y., Shen Y., Jin Z. (2021). Synergetic modification of waxy maize starch by dual-enzyme to lower the in vitro digestibility through modulating molecular structure and malto-oligosaccharide content. Int. J. Biol. Macromol..

[19] Gui Y., Zou F., Li J., Tang J., Guo L., Cui B. (2021). Corn starch modification during endogenous malt amylases: The impact of synergistic hydrolysis time of α-amylase and β-amylase and limit dextrinase. Int. J. Biol. Macromol..

[20] Evans D.E., Dambergs R., Ratkowsky D., Li C., Harasymow S., Roumeliotis S., Eglinton J.K. (2010). Refining the Prediction of Potential Malt Fermentability by Including an Assessment of Limit Dextrinase Thermostability and Additional Measures of Malt Modification, Using Two Different Methods for Multivariate Model Development. J. Inst. Brew..

[21] Park K.-H., Park J.-H., Lee S., Yoo S.-H., Kim J.-W., Park K.-H. (2008). Enzymatic Modification of Starch for Food Industry. Carbohydrate-Active Enzymes.

[22] Cui Y., Wang W., Gao W., Shi M., Liu S., Liu C., Zheng M., Liu M., Liu H., Liu J. (2024). Effect of static magnetic field pretreatment on the structure and oil absorption properties of normal maize starch. Food Hydrocoll..

[23] Yashiro K., Sato S., Kondo Y., Nakamura Y., Yano H., Koda T., Nishioka A., Takashima Y., Matsuba G. (2024). Relationship between the nanometer-scale structures of amylopectin molecules and temperature dependence of internal structures of starch granules in endosperm of starch branching enzyme 2b (be2b) allelic mutant lines from japonica rice. Results Chem..

[24] Gu F., Gong B., Gilbert R.G., Yu W., Li E., Li C. (2019). Relations between changes in starch molecular fine structure and in thermal properties during rice grain storage. Food Chem..

[25] Qiao J., Jia M., Niu J., Zhang Z., Xing B., Liang Y., Li H., Zhang Y., Ren G., Qin P. (2024). Amylopectin chain length distributions and amylose content are determinants of viscoelasticity and digestibility differences in mung bean starch and proso millet starch. Int. J. Biol. Macromol..

[26] Ren J., Chen S., Li C., Gu Z., Cheng L., Hong Y., Li Z. (2020). A two-stage modification method using 1,4-α-glucan branching enzyme lowers the in vitro digestibility of corn starch. Food Chem..

[27] Zhong Y., Keeratiburana T., Kain Kirkensgaard J.J., Khakimov B., Blennow A., Hansen A.R. (2021). Generation of short-chained granular corn starch by maltogenic α-amylase and transglucosidase treatment. Carbohydr. Polym..

[28] Chung J.H., Han J.A., Yoo B., Seib P.A., Lim S.T. (2008). Effects of molecular size and chain profile of waxy cereal amylopectins on paste rheology during retrogradation. Carbohydr. Polym..

[29] Chang Y.-H., Lin J.-H. (2007). Effects of molecular size and structure of amylopectin on the retrogradation thermal properties of waxy rice and waxy cornstarches. Food Hydrocoll..

[30] Morikawa K., Nishinari K. (2000). Effects of concentration dependence of retrogradation behaviour of dispersions for native and chemically modified potato starch. Food Hydrocoll..

[31] Chen L., Ren F., Zhang Z., Tong Q., Rashed M.M.A. (2015). Effect of pullulan on the short-term and long-term retrogradation of rice starch. Carbohydr. Polym..

[32] Lin J.-H., Lii C.-y., Chang Y.-H. (2005). Change of granular and molecular structures of waxy maize and potato starches after treated in alcohols with or without hydrochloric acid. Carbohydr. Polym..

[33] Di Y., Na R., Xia H., Wang Y., Li F. (2023). Irradiation effects on characteristics and ethanol fermentation of maize starch. Int. J. Biol. Macromol..

[34] Chen L., McClements D.J., Yang T., Ma Y., Ren F., Tian Y., Jin Z. (2021). Effect of annealing and heat-moisture pretreatments on the oil absorption of normal maize starch during frying. Food Chem..

[35] Takata H., Takaha T., Okada S., Hizukuri S., Takagi M., Imanaka T. (1996). Structure of the cyclic glucan produced from amylopectin by Bacillus stearothermophilus branching enzyme. Carbohydr. Res..

[36] Liu L., Jiang X., Chen Y., Yaqoob S., Xiu L., Liu H., Zheng M., Cai D., Liu J. (2024). Germination-induced modifications of starch structure, flour-processing characteristics, and in vitro digestive properties in maize. Food Chem. X.

[37] Zhong Y., Herburger K., Kirkensgaard J.J.K., Khakimov B., Hansen A.R., Blennow A. (2021). Sequential maltogenic α-amylase and branching enzyme treatment to modify granular corn starch. Food Hydrocoll..

[38] Xia C., Zhong L., Wang J., Zhang L., Chen X., Ji H., Ma S., Dong W., Ye X., Huang Y. (2021). Structural and digestion properties of potato starch modified using an efficient starch branching enzyme AqGBE. Int. J. Biol. Macromol..

[39] Rahaman A., Kumari A., Zeng X.-A., Adil Farooq M., Siddique R., Khalifa I., Siddeeg A., Ali M., Faisal Manzoor M. (2021). Ultrasound based modification and structural-functional analysis of corn and cassava starch. Ultrason. Sonochemistry.

[40] Li X., Miao M., Jiang H., Xue J., Jiang B., Zhang T., Gao Y., Jia Y. (2014). Partial branching enzyme treatment increases the low glycaemic property and α-1,6 branching ratio of maize starch. Food Chem..

[41] Cheetham N.W.H., Tao L. (1998). Variation in crystalline type with amylose content in maize starch granules: An X-ray powder diffraction study. Carbohydr. Polym..

[42] Jo A.R., Kim H.R., Choi S.J., Lee J.S., Chung M.N., Han S.K., Park C.-S., Moon T.W. (2016). Preparation of slowly digestible sweet potato Daeyumi starch by dual enzyme modification. Carbohydr. Polym..

[43] Chen Y., Dai G., Gao Q. (2020). Preparation and properties of granular cold-water-soluble porous starch. Int. J. Biol. Macromol..

[44] Hajihashemi Z., Nasirpour A., Scher J., Desobry S. (2014). Interactions among lactose, β-lactoglobulin and starch in co-lyophilized mixtures as determined by Fourier Transform Infrared Spectroscopy. J. Food Sci. Technol..

[45] Wang X., Leng X., Zhang G. (2020). The loosening effect of tea polyphenol on the structure of octenyl succinic anhydride modified waxy maize starch. Food Hydrocoll..

[46] Bai Y., Shi Y.-C., Herrera A., Prakash O. (2011). Study of octenyl succinic anhydride-modified waxy maize starch by nuclear magnetic resonance spectroscopy. Carbohydr. Polym..

[47] Guo L. (2018). Sweet potato starch modified by branching enzyme, β-amylase and transglucosidase. Food Hydrocoll..

[48] Guo W., Yang L., Shi X., Cong X., Cheng S., Li L., Cheng H. (2024). Effects of color protection and enzymatic hydrolysis on the microstructure, digestibility, solubility and swelling degree of chestnut flour. Food Chem. X.

[49] Hanashiro I., Abe J.-i., Hizukuri S. (1996). A periodic distribution of the chain length of amylopectin as revealed by high-performance anion-exchange chromatography. Carbohydr. Res..

[50] Hanashiro I., Tagawa M., Shibahara S., Iwata K., Takeda Y. (2002). Examination of molar-based distribution of A, B and C chains of amylopectin by fluorescent labeling with 2-aminopyridine. Carbohydr. Res..

[51] Zhong Y., Liu L., Qu J., Blennow A., Hansen A.R., Wu Y., Guo D., Liu X. (2020). Amylose content and specific fine structures affect lamellar structure and digestibility of maize starches. Food Hydrocoll..

[52] Shin H.J., Choi S.J., Park C.S., Moon T.W. (2010). Preparation of starches with low glycaemic response using amylosucrase and their physicochemical properties. Carbohydr. Polym..

[53] Kim E.-J., Ryu S.-I., Bae H.-A., Huong N.T., Lee S.-B. (2008). Biochemical characterisation of a glycogen branching enzyme from Streptococcus mutans: Enzymatic modification of starch. Food Chem..

[54] Ren J., Li Y., Li C., Gu Z., Cheng L., Hong Y., Li Z. (2017). Pasting and thermal properties of waxy corn starch modified by 1,4-α-glucan branching enzyme. Int. J. Biol. Macromol..

[55] Fan H., Chen Z., Ma R., Wen Y., Li H., Wang J., Sun B. (2022). Effect of alkyl chain length and amylose/amylopectin ratio on the structure and digestibility of starch-alkylresorcinols inclusion complexes. Food Hydrocoll..

[56] Obadi M., Qi Y., Xu B. (2023). High-amylose maize starch: Structure, properties, modifications and industrial applications. Carbohydr. Polym..

[57] Lin L., Cai C., Gilbert R.G., Li E., Wang J., Wei C. (2016). Relationships between amylopectin molecular structures and functional properties of different-sized fractions of normal and high-amylose maize starches. Food Hydrocoll..

[58] Li Y., Hu A., Zheng J., Wang X. (2019). Comparative studies on structure and physiochemical changes of millet starch under microwave and ultrasound at the same power. Int. J. Biol. Macromol..

[59] Ma Y., Zhang W., Pan Y., Ali B., Xu D., Xu X. (2021). Physicochemical, crystalline characterization and digestibility of wheat starch under superheated steam treatment. Food Hydrocoll..

[60] Wang Y., Bai Y., Ji H., Dong J., Li X., Liu J., Jin Z. (2022). Insights into rice starch degradation by maltogenic α–amylase: Effect of starch structure on its rheological properties. Food Hydrocoll..

[61] Liu M., Wang X., Li Y., Jin D., Jiang Y., Fang Y., Lin Q., Ding Y. (2024). Effects of OSA-starch-fatty acid interactions on the structural, digestibility and release characteristics of high amylose corn starch. Food Chem..

[62] Wu K., Li C., Li Z., Gu Z., Ban X., Hong Y., Cheng L., Kong H. (2024). Enzymatic modification lowers syneresis in corn starch gels during freeze–thaw cycles through 1,4-α-glucan branching enzyme. Int. J. Biol. Macromol..

[63] Zhang K., Zhao D., Guo D., Tong X., Zhang Y., Wang L. (2021). Physicochemical and digestive properties of A- and B-type granules isolated from wheat starch as affected by microwave-ultrasound and toughening treatment. Int. J. Biol. Macromol..

[64] Ye J., Hu X., Luo S., McClements D.J., Liang L., Liu C. (2018). Effect of endogenous proteins and lipids on starch digestibility in rice flour. Food Res. Int..

[65] Han X., Wen H., Luo Y., Yang J., Xiao W., Ji X., Xie J. (2021). Effects of α-amylase and glucoamylase on the characterization and function of maize porous starches. Food Hydrocoll..

